# Curiouser and Curiouser: The Macrocyclic Lactone, Abamectin, Is also a Potent Inhibitor of Pyrantel/Tribendimidine Nicotinic Acetylcholine Receptors of Gastro-Intestinal Worms

**DOI:** 10.1371/journal.pone.0146854

**Published:** 2016-01-11

**Authors:** Melanie Abongwa, Samuel K. Buxton, Alan P. Robertson, Richard J. Martin

**Affiliations:** Department of Biomedical Sciences, College of Veterinary Medicine, Iowa State University, Ames, Iowa, United States of America; UMASS Medical School, UNITED STATES

## Abstract

Nematode parasites may be controlled with drugs, but their regular application has given rise to concerns about the development of resistance. Drug combinations may be more effective than single drugs and delay the onset of resistance. A combination of the nicotinic antagonist, derquantel, and the macrocyclic lactone, abamectin, has been found to have synergistic anthelmintic effects against gastro-intestinal nematode parasites. We have observed in previous contraction and electrophysiological experiments that derquantel is a potent selective antagonist of nematode parasite muscle nicotinic receptors; and that abamectin is an inhibitor of the same nicotinic receptors. To explore these inhibitory effects further, we expressed muscle nicotinic receptors of the nodular worm, *Oesophagostomum dentatum* (Ode-UNC-29:Ode-UNC-63:Ode-UNC-38), in *Xenopus* oocytes under voltage-clamp and tested effects of abamectin on pyrantel and acetylcholine responses. The receptors were antagonized by 0.03 μM abamectin in a non-competitive manner (reduced *R*_*max*_, no change in *EC*_*50*_). This antagonism increased when abamectin was increased to 0.1 μM. However, when we increased the concentration of abamectin further to 0.3 μM, 1 μM or 10 μM, we found that the antagonism decreased and was less than with 0.1 μM abamectin. The bi-phasic effects of abamectin suggest that abamectin acts at two allosteric sites: one high affinity negative allosteric (NAM) site causing antagonism, and another lower affinity positive allosteric (PAM) site causing a reduction in antagonism. We also tested the effects of 0.1 μM derquantel alone and in combination with 0.3 μM abamectin. We found that derquantel on these receptors, like abamectin, acted as a non-competitive antagonist, and that the combination of derquantel and abamectin produced greater inhibition. These observations confirm the antagonistic effects of abamectin on nematode nicotinic receptors in addition to GluCl effects, and illustrate more complex effects of macrocyclic lactones that may be exploited in combinations with other anthelmintics.

## Introduction

Gastro-intestinal nematode parasite infections of both humans and animals are a global public health problem. These infections cause livestock production losses, morbidity, and if left uncontrolled, may result in death [1]. In the absence of effective sanitation and vaccines, control of nematode parasite infections is achieved by the use of anthelmintic drugs, many of which act on ligand-gated ion channels (LGICs) [2–4]. The nicotinic acetylcholine receptor (nAChR) is a prototypic pentameric LGIC. Different subunit combinations or different stoichiometry of the same nAChR subunits can give rise to multiple receptor subtypes with different pharmacological properties [5, 6]. There are a limited number of anthelmintics which are currently approved for use [2]. Those that act on nAChRs include: imidazothiazoles (levamisole) [7], tetrahydropyrimidine derivatives (pyrantel, oxantel, morantel) [8], spiroindoles (derquantel) [9], and tribendimidine [10]. The macrocyclic lactones (ivermectin, abamectin, moxidectin) act on glutamate-gated chloride channels (GluCls) [11]; the benzimidazoles (albendazole, mebendazole) act on β-tubulin [12]; and piperazine acts on GABA receptors [13]. Other more recent drugs include the amino-acetonitrile derivatives (monepantel) which act on LGICs receptors comprised of DEG-3/DES-2 nAChR subunits [14]; and the cyclooctadepsipeptide (emodepside) whose mode of action is understood to be on SLO-1 potassium channels [15, 16] and latrophilin receptors [17].

**Fig 1:**
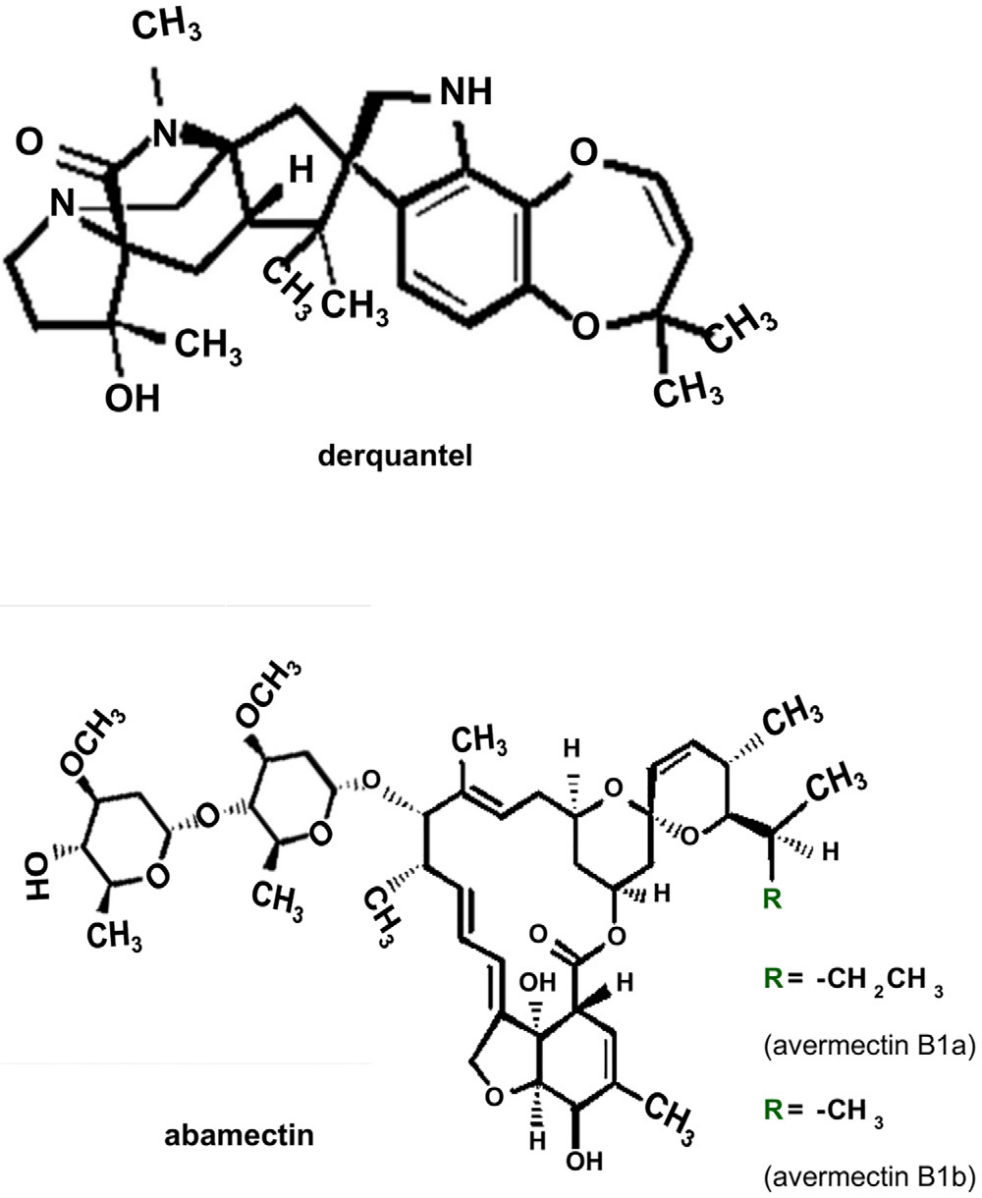
Structures of derquantel and abamectin. **A**: Structure of derquantel. **B**: Structure of abamectin. Abamectin is a mixture of avermectin B1a and avermectin B1b. Avermectin B1a differs from avermectin B1b by a functional group at the ‘R’ position, and makes up more than 80% of abamectin, while avermectin B1b makes up less than 20% of abamectin.

The increased use of anthelmintics has led to the development of resistance in both animal and human nematode parasites [18]. Resistance to anthelmintics presents a serious drawback in the control of nematode parasite infections and is an increasing medical concern [19]. Combination therapy involving anthelmintic drugs of different classes is an important approach that may slow the development of anthelmintic drug resistance. The simultaneous development of resistance to two drugs is less likely than the development of resistance to one drug alone; hence combinations are predicted to slow the development of resistance [20]. Startect^®^ (derquantel and abamectin combination, Fig 1) is a more recently introduced anthelmintic combination that is marketed for use in sheep, but it has been shown to have effects on nematode parasites of other host species such as *Ascaris suum* of pigs [21]. In this study, we investigated the effects of derquantel alone, abamectin alone, and a combination of derquantel and abamectin on a nAChR subtype from the nematode parasite *Oesophagostomum dentatum*. This subtype is comprised of *Ode*-UNC-29:*Ode*-UNC-63:*Ode*-UNC-38 (29-63-38) nAChR subunits. Other pharmacologically characterized nAChR subtypes of *O*. *dentatum* are *Ode*-UNC-29:*Ode*-UNC-63 (pyrantel-sensitive nAChR), *Ode*-UNC-29:*Ode*-UNC-63:*Ode*-ACR-8 (acetylcholine-sensitive nAChR), and *Ode*-UNC-29:*Ode*-UNC-63:*Ode*-UNC-38:*Ode*-ACR-8 (levamisole-sensitive nAChR) [5]. We chose *O*. *dentatum* parasite for further investigation because the worm is easily maintained and passaged, and it is a Clade V nematode, just like the model free-living nematode, *Caenorhabditis elegans* [22]. *O*. *dentatum* is a common nodule worm in pigs, very similar to other *Oesophagostomum* species which infect humans, most notably in northern Togo and Ghana [23]. The *O*. *dentatum* nAChR subtype, *Ode*-UNC-29:*Ode*-UNC-63:*Ode*-UNC-38 used in this study is preferentially activated by pyrantel and tribendimidine, and it is antagonized in a non-competitive manner by derquantel [5]. We show here that abamectin is a potent antagonist of these nAChRs, and that a combination of derquantel and abamectin has greater effects than derquantel or abamectin used alone. These additive effects support the continued use of the derquantel and abamectin combination for the control of nematode parasites.

## Materials and Methods

Experiments described in this study have been conducted according to US national and international welfare guidelines. The Office for Responsible Research at Iowa State University, IACUC Log #3-2-5134-s specifically approved this study and granted permission for the culture of the *Oesophagostomum* parasites. There was no animal suffering or surgery required.

### Cloning of nAChR subunits from *O*. *dentatum* and ancillary factors from *Haemonchus contortus*

*O*. *dentatum* nAChR subunits and *H*. *contortus* ancillary factors used in this research study have been previously cloned and reported [5].

### Expression of *Ode*-UNC-29:*Ode*-UNC-63:*Ode*-UNC-38 in *Xenopus laevis* oocytes

Defolliculated *X*. *laevis* oocytes were purchased from Ecocyte Bioscience (Austin, TX, USA). Oocyte microinjection was done using a Drummond nanoject II microinjector (Drummond Scientific, PA, and USA). 1.8 ng of each subunit cRNA (*Ode-unc-29*, *Ode-unc-63* and *Ode-unc-38*) and ancillary factor cRNA (*Hco-ric-3*, *Hco-unc-50* and *Hco-unc-74*) in a total volume of 36 nL in RNAse-free water were microinjected into the animal pole of the oocytes. Once micro-injected, the oocytes were transferred to a sterile 96-well culture plate (one oocyte/well) containing 200 μL incubation solution (100 mM NaCl, 2 mM KCl, 1.8 mM CaCl_2_.2H_2_0, 1 mM MgCl_2_.6H_2_0, and 5 mM HEPES, 2.5 mM Na pyruvate, 100 U/mL penicillin and 100 μg/mL streptomycin, pH 7.5) in each well. The oocytes were incubated at 19°C for 2–5 days, with a daily change of incubation solution.

### Two-microelectrode voltage-clamp (TEVC) electrophysiology

We followed methods we have described in detail before [5]. Briefly, 100 μM BAPTA-AM was added to the oocytes in incubation media approximately 3 hours prior to electrophysiological recordings from the oocytes. TEVC recordings were carried out by impaling the oocytes with two microelectrodes; a voltage sensing electrode, Vm, and a current injecting electrode, Im, were used to inject the current required to hold the membrane at the set voltage. The microelectrodes were pulled with a Flaming/Brown horizontal electrode puller (Model P-97, Sutter Instruments), filled with 3 M potassium chloride and the microelectrode tips carefully broken with a piece of tissue paper to achieve a low resistance of 2–5 MΩ in recording solution (100 mM NaCl, 2.5 mM KCl, 1 mM CaCl_2_.2H_2_O and 5 mM HEPES, pH 7.3). Current/voltage signals were amplified by an AxoClamp 2B amplifier (Molecular Devices, CA, USA). The amplified signals were converted from analog to digital format by a Digidata 1322A digitizer (Molecular Devices, CA, USA) and finally acquired on a desktop computer with the Clampex 9.2 data acquisition software (Molecular Devices, CA, USA).

### Drugs

Acetylcholine (ach) and pyrantel (pyr) were purchased from Sigma-Aldrich (St Louis, MO, USA), while derquantel (der) and abamectin (aba) were gifts from Zoetis (Kalamazoo, MI). Acetylcholine and pyrantel were dissolved in recording solution. Derquantel and abamectin were dissolved in DMSO and added to recording solution at a final concentration of ≤ 0.01% DMSO.

### Drug applications

Acetylcholine which is a natural agonist of many nAChRs served for normalization and as a control in our experiments. Each agonist concentration was applied for 10 s, with sufficient time allowed for wash off between drug applications. For every oocyte recording, 100 μM acetylcholine was applied initially and this current response was measured, subsequent responses were measured and normalized to this acetylcholine current. To generate control agonist concentration-response plots, acetylcholine was applied at different concentrations between 0.1–100 μM or pyrantel was applied at different concentrations between 0.03–10 μM. Higher concentrations of pyrantel were not tested because pyrantel acts as an open channel blocker at high concentrations [24]. For the antagonist experiments, a control 100 μM acetylcholine was also first applied for 10s, and then followed by a 2 min or 10 min challenge with the antagonist (derquantel and/or abamectin), and a final application of the agonist (pyrantel) in the continued presence of the antagonist.

### Data analysis

We used Clampfit 9.2 (Molecular Devices, CA, and USA) to measure peak current responses and normalized them to the first 100 μM acetylcholine responses. GraphPad Prism 5.0 software (GraphPad Software Inc., USA) was used to analyze the data, and results were expressed as mean ± S.E.M. Concentration-response data points were fitted with the Hill equation as previously described: where *R*_*max*_ is the maximum response % relative to the control 100 μM ach response; the *EC*_*50*_ is the concentration producing the half-maximum response and *n*_*H*_ is the slope factor or Hill coefficient [25]. We used the unpaired two-tailed Student’s t-test to test for statistical significance and a p value < 0.05 was considered significant.

The Bliss additive effect dose-response relationship for pyrantel current responses was calculated as previously described [26] to predict the linear additive effects of derquantel and abamectin on the *Ode*-UNC-29:*Ode*-UNC-63:*Ode*-UNC-38 nAChR. To do so, we determined: the normalized mean current responses to each control pyrantel concentration (*CR[pyr]*); the mean current responses to each concentration of pyrantel in the presence of derquantel (derquantel effect) and; the mean current responses to each concentration of pyrantel in the presence of abamectin (abamectin effect). We then used the fractional inhibition (mean reduction in response/*CR[pyr]*) produced by derquantel alone, *Fd*, and abamectin alone, *Fa*, at each pyrantel concentration to determine the additive effect. We used *Fa(1-Fd)* to denote the fractional inhibition produced by abamectin when derquantel is already present, and *Fd*+*Fa(1-Fd)* to denote the fractional inhibition produced by the combination of derquantel and abamectin. Lastly, the normalized additive response was calculated as:

CR[pyr]−CR[pyr]{Fd+Fa(1−Fd)}

The difference between the observed and calculated additive effects for the combination of derquantel and abamectin were tested for statistical significance using the paired two-tailed Student’s t-test, taking p < 0.05 as significant.

## Results

### *Ode*-UNC-29:*Ode*-UNC-63:*Ode*-UNC-38 forms a pyrantel-sensitive nAChR

**Fig 2:**
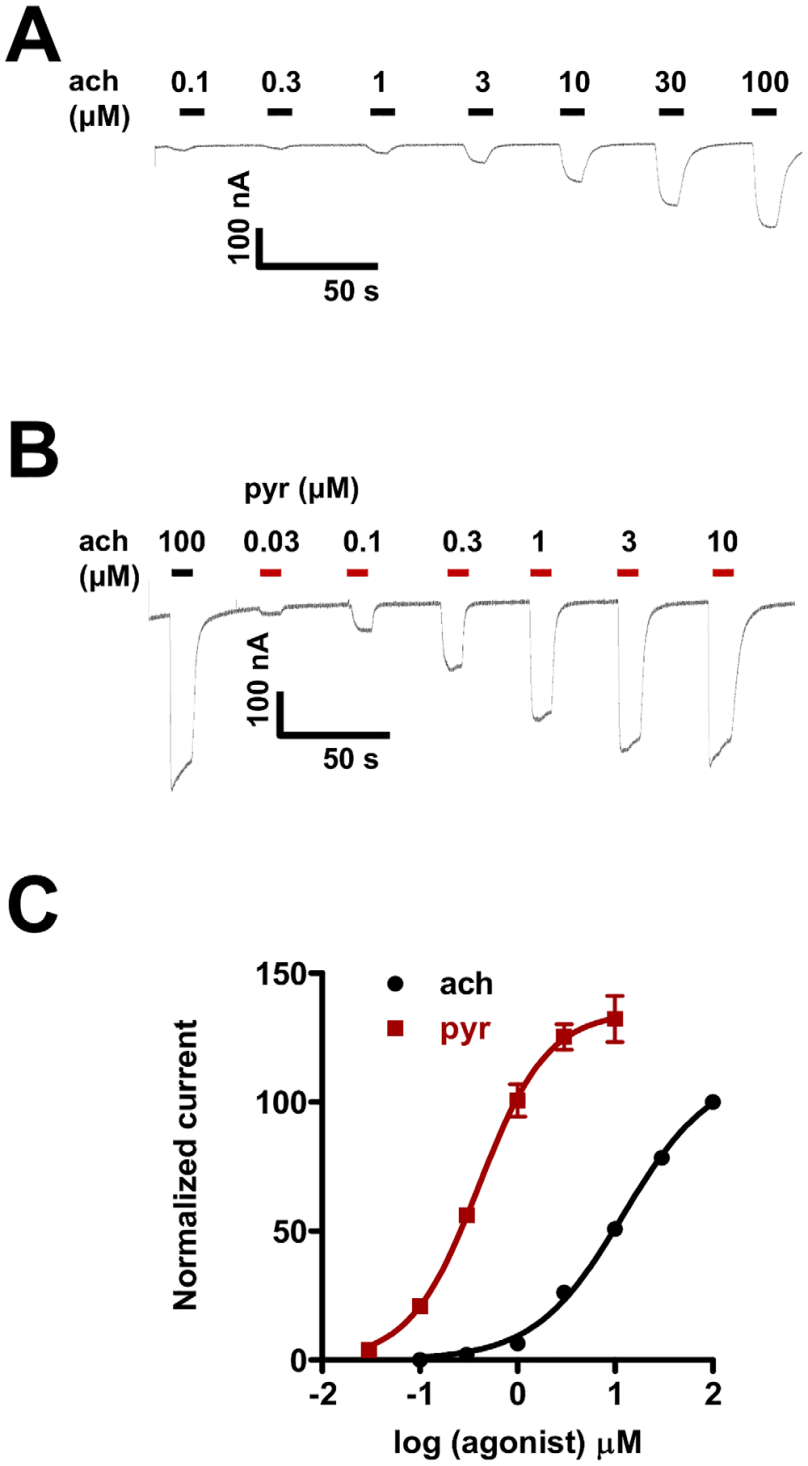
Acetylcholine and pyrantel concentration-response relationships for the *Ode*-UNC-29:*Ode*-UNC-63:*Ode*-UNC-38 receptor. **A**: Representative trace (inward currents, holding potential -60mV, from oocytes expressing *Ode*-UNC-29:*Ode*-UNC-63:*Ode*-UNC-38) following 10 seconds applications of different acetylcholine concentrations, from 0.1 μM to 100 μM. **B**: Representative trace (inward currents, holding potential -60mV, from oocytes expressing *Ode*-UNC-29:*Ode*-UNC-63:*Ode*-UNC-38) of the 10 seconds application of different pyrantel concentrations, from 0.03 μM to 10 μM. An initial 10 seconds application of 100 μM acetylcholine served as the control. **C**: Concentration-response plots for acetylcholine (n = 4, black) and pyrantel (n = 6, red). Results were normalized to 100 μM acetylcholine current responses and expressed as mean ± S.E.M.

Representative traces (inward currents from oocytes expressing *Ode*-UNC-29:*Ode*-UNC-63:*Ode*-UNC-38) produced in response to different concentrations of acetylcholine and pyrantel are shown in Fig 2A and 2B respectively. Fig 2C shows acetylcholine and pyrantel concentration-response relationships for *Ode*-UNC-29:*Ode*-UNC-63:*Ode*-UNC-38. The *EC*_*50*_ and maximum response (*R*_*max*_) values for acetylcholine were 13.0 ± 1.6 μM and 114.3 ± 4.3%, n = 4. The *EC*_*50*_ and *R*_*max*_ values for pyrantel were 0.4 ± 0.0 μM and 135.5 ± 7.9%, n = 6 (S1 Table). Our results showed the *EC*_*50*_ for pyrantel to be significantly smaller (p < 0.001) than the *EC*_*50*_ for acetylcholine, but showed no significant difference (p > 0.05) in *R*_*max*_ between acetylcholine and pyrantel. Based on these *EC*_*50*_ values, pyrantel is 32.5 times more potent than acetylcholine on the *Ode*-UNC-29:*Ode*-UNC-63:*Ode*-UNC-38 nAChR. Also there was a slightly steeper Hill slope (*nH*) for pyrantel than acetylcholine (*nH* = 1.2 ± 0.1, n = 6 for pyrantel; *nH* = 1.0 ± 0.1, n = 4 for acetylcholine; p < 0.05). We used pyrantel rather than acetylcholine for most subsequent experiments because of its potency and because it is a more selective agonist than acetylcholine for this nAChR subtype [5].

### Effects of abamectin on *Ode*-UNC-29:*Ode*-UNC-63:*Ode*-UNC-38

**Fig 3:**
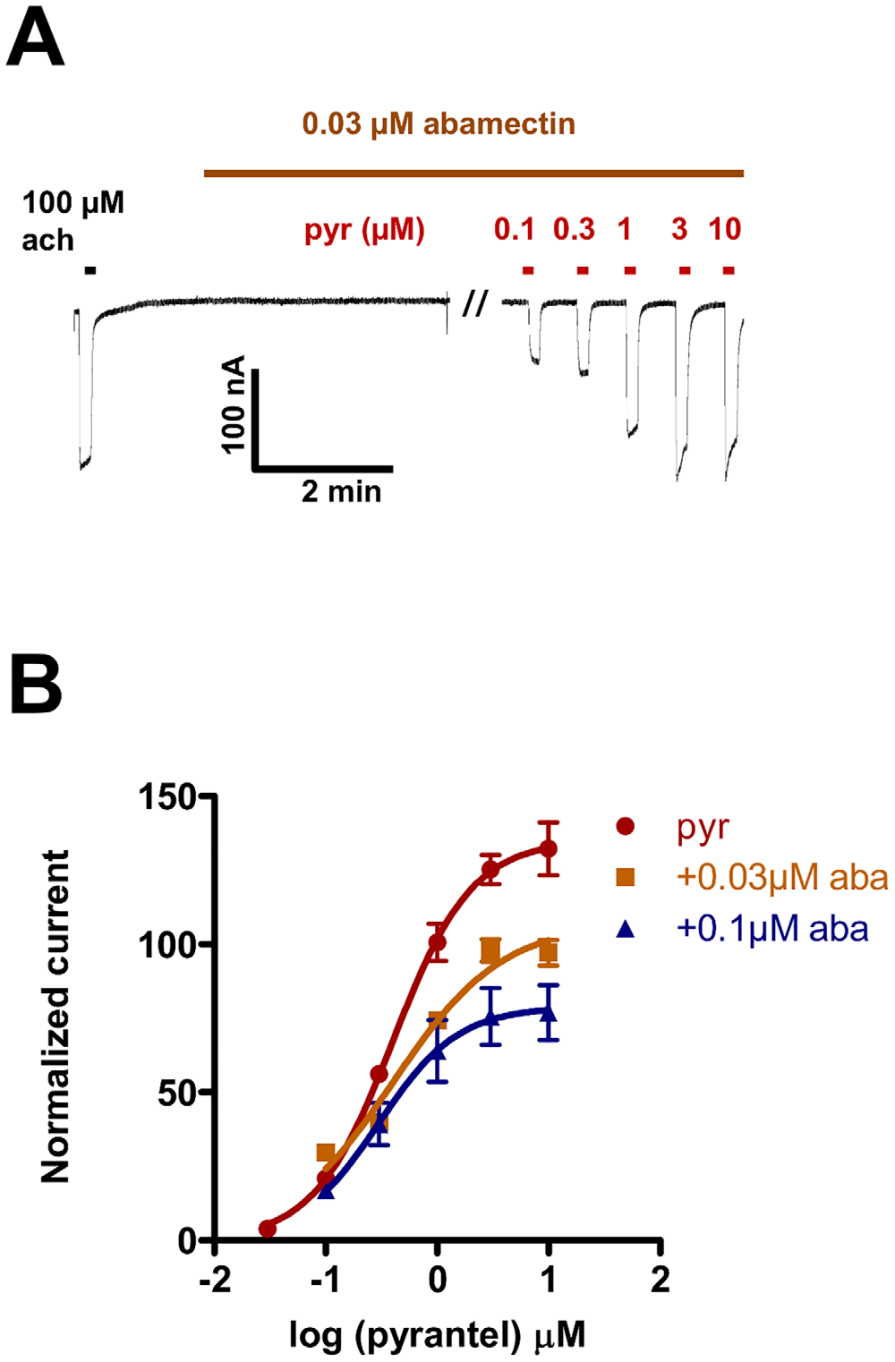
Pyrantel concentration-response relationships in the presence of 0.03 μM and 0.1 μM abamectin. **A**: Representative trace (inward currents, holding potential -60mV, from oocytes expressing *Ode*-UNC-29:*Ode*-UNC-63:*Ode*-UNC-38) of the 10 seconds application of the control 100 μM acetylcholine, followed by a 10 minutes application of abamectin (0.03 μM) and finally, a 10 seconds application of different pyrantel concentrations in the continued presence of abamectin. **B**: Concentration-response plots for pyrantel in the absence (n = 6, red) and presence of 0.03 μM abamectin (n = 5, orange) and 0.1 μM abamectin (n = 6, dark blue). Results were normalized to 100 μM acetylcholine current responses and expressed as mean ± S.E.M. Notice 0.03 μM abamectin caused an inhibition of current responses to pyrantel, and this inhibition was greater with 0.1 μM abamectin.

We investigated pyrantel concentration-response relationships on *Ode*-UNC-29:*Ode*-UNC-63:*Ode*-UNC-38 in the presence of 0.03 μM and 0.1 μM abamectin. A representative trace is shown in Fig 3A. The concentration-response plots, Fig 3B, show abamectin to be an antagonist of *Ode*-UNC-29:*Ode*-UNC-63:*Ode*-UNC-38. The *EC*_*50*_ and *R*_*max*_ values were: 0.4 ± 0.0 μM and 135.5 ± 7.9%, n = 6 for pyrantel in the absence of abamectin; 0.4 ± 0.0 μM and 107.3 ± 4.7%, n = 5 for pyrantel in the presence of 0.03 μM abamectin; and 0.3 ± 0.0 μM and 80.6 ± 8.4%, n = 6 for pyrantel in the presence of 0.1 μM abamectin. There was no significant difference (p > 0.05) in the *EC*_50s_ between pyrantel in the absence and presence of 0.03 μM or 0.1 μM abamectin. But, there was a significant difference (p < 0.05) in *R*_*max*_ for pyrantel in the absence and presence of 0.03 μM abamectin, and this difference was greater (p < 0.001) in the presence of 0.1 μM abamectin. Hence, abamectin did not cause a significant change in *EC*_*50*_, but did cause a significant reduction in *R*_*max*,_ showing that abamectin acts as a non-competitive antagonist of the *Ode*-UNC-29:*Ode*-UNC-63:*Ode*-UNC-38 nAChR.

**Fig 4:**
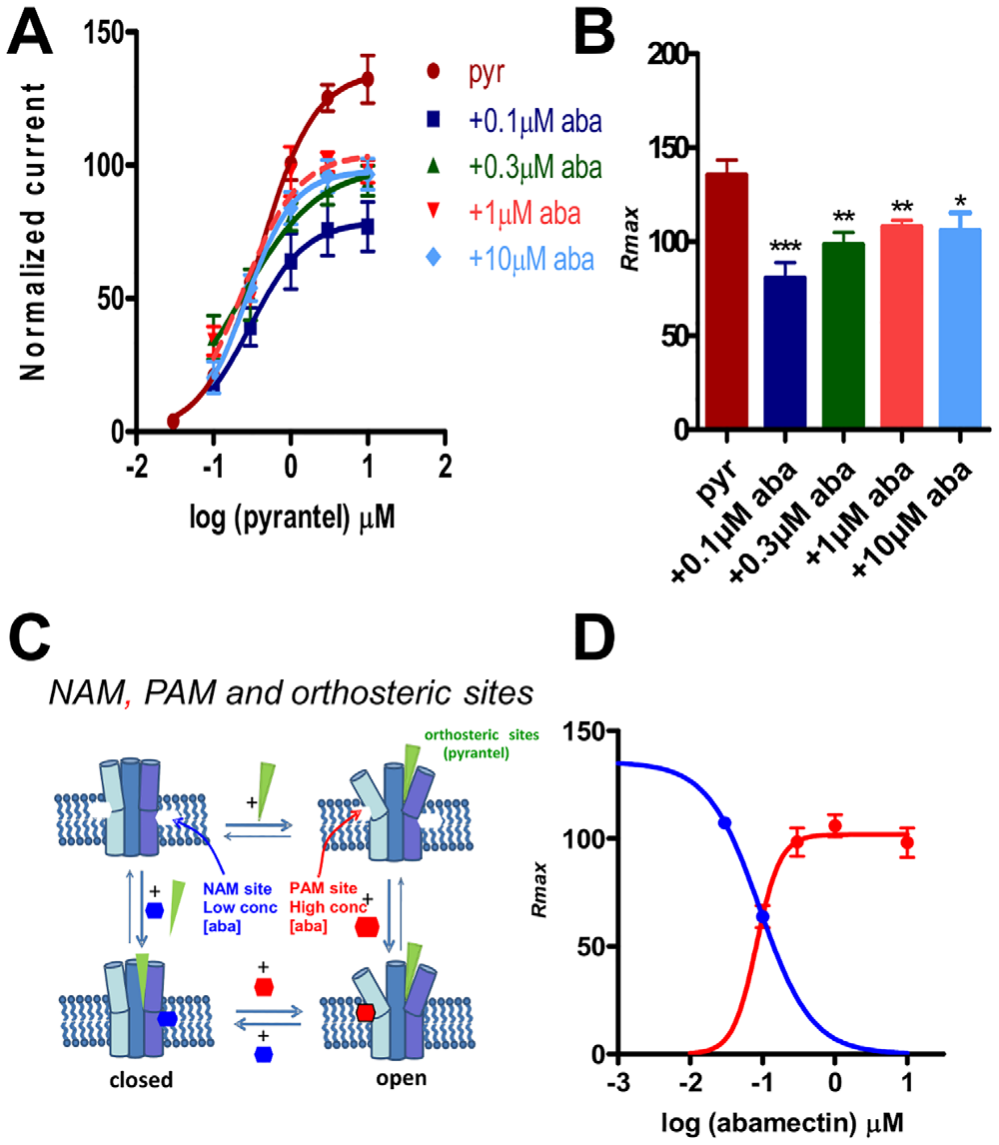
Effects of increasing concentrations of abamectin (above 0.1 μM) on the pyrantel concentration-response plots. **A**: Concentration-response plots for pyrantel in the absence (n = 6, red) and presence of 0.1 μM abamectin (n = 6, dark blue); 0.3 μM abamectin (n = 5, dark green); 1 μM abamectin (n = 6, pink); 10 μM abamectin (n = 5, light blue). Results were normalized to 100 μM acetylcholine current responses and expressed as mean ± S.E.M. Increasing the concentration of abamectin from 0.1 μM to 0.3, 1 and 10 μM rather caused a reduction in the inhibition instead of a potentiation. **B**: Bar chart showing the mean ± S.E.M of the maximum current responses (*R*_*max*_) for pyrantel and the different abamectin concentrations. *R*_*max*_ for 0.1 μM abamectin (n = 6, dark blue), 0.3 μM abamectin (n = 5, dark green), 1 μM abamectin (n = 6, pink) and 10 μM abamectin (n = 5, light blue) were significantly lower than *R*_*max*_ for pyrantel alone (n = 6, red). * p < 0.05, ** p < 0.01 and *** p < 0.001, unpaired two-tailed student t-test. **C**: Model of ligand sites of action. Pyrantel binds to the orthosteric sites opening the channel. Low concentrations of abamectin (0.03 and 0.1 μM) bind to a negative allosteric site (NAM) in the lipid phase of the channel, inhibiting opening. Higher concentrations of abamectin (0.3, 1 and 10 μM) bind to a positive allosteric site (PAM) increasing opening. **D**: Abamectin *R*_*max*_ inhibition (blue curve) and inhibition reduction (red curve) dose response plots. The data points for inhibition used data from 0.03 μM and 0.1 μM abamectin from Fig 3. The data points for inhibition reduction used data from Fig 4B.

When we increased the concentration of abamectin from 0.1 μM to 0.3 μM, Fig 4A, we noticed that the *EC*_*50*_ for pyrantel in the presence of 0.3 μM abamectin remained unchanged: 0.3 ± 0.1 μM, n = 5 (p > 0.05). Although not statistically significant (p > 0.05), the mean value for the *R*_*max*_ for pyrantel in the presence of 0.1 μM abamectin (80.6 ± 8.4, n = 6) appeared smaller than *R*_*max*_ in the presence of 0.3 μM abamectin (98.4 ± 6.5, n = 5). This suggested that the inhibitory effects of lower concentrations of abamectin can be greater than those of higher concentrations. This reversed inhibitory effect was tested and confirmed when we increased the concentration of abamectin from 0.3 μM to 1 μM and 10 μM. While the *EC*_*50*_ values did not change (0.3 ± 0.1 μM, n = 6 in the presence of 1 μM abamectin, and 0.4 ± 0.1 μM, n = 5 in the presence of 10 μM abamectin), the *R*_*max*_ values increased to 108.0 ± 3.5%, n = 6 in the presence of 1 μM abamectin, and 106.0 ± 9.4%, n = 5 in the presence of 10 μM abamectin. We point out that the effects of abamectin were reversible on washing, although the current responses did not return to control responses even after a 10 mins wash. S2 Table summarizes the *EC*_*50*_, *R*_max_, *n*_*H*_ and n numbers for these experiments. Interestingly, when the *EC*_50s_ for pyrantel alone are compared with that for pyrantel in the presence of 0.1 μM, 0.3 μM, 1 μM or 10 μM abamectin, there were no significant differences in the *EC*_*50*_ values. In contrast however, the *R*_*max*_ values for pyrantel in the presence of 0.1 μM, 0.3 μM, 1 μM and 10 μM abamectin were significantly less than the *R*_*max*_ for pyrantel alone, Fig 4B. We emphasize again that the *R*_*max*_ for pyrantel in the presence of abamectin concentrations ≥0.3 μM were greater than the *R*_*max*_ for pyrantel in the presence of 0.1 μM abamectin, with the difference being statistically significant (p < 0.05) for 1 μM. These observations show that abamectin has a bi-phasic effect, suggesting two allosteric sites of action: a high affinity site causing antagonism, and a lower affinity site causing a reduction in the antagonism, Fig 4C and 4D.

### Effects of derquantel and abamectin combination on *Ode*-UNC-29:*Ode*-UNC-63:*Ode*-UNC-38

**Fig 5:**
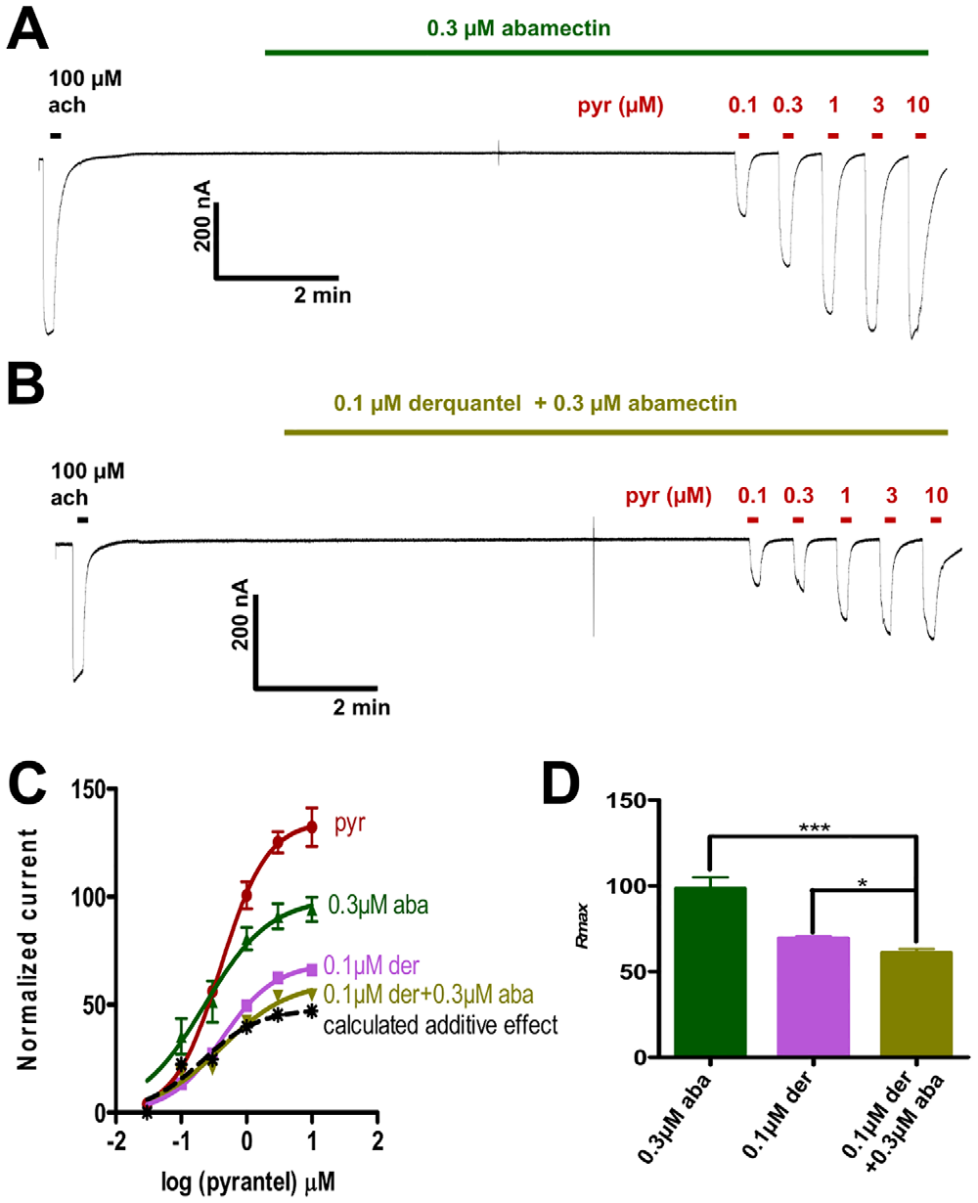
Effects of 0.1 μM derquantel alone, 0.3 μM abamectin alone, and derquantel and abamectin combination (0.1 μM derquantel + 0.3 μM abamectin) on the pyrantel concentration-response plots. **A**: Representative trace (inward currents from oocytes expressing *Ode*-UNC-29:*Ode*-UNC-63:*Ode*-UNC-38) of the 10 seconds application of the control 100 μM acetylcholine, followed by a 10 minutes application of 0.3 μM abamectin and finally, a 10 seconds application of different pyrantel concentrations in the continued presence of abamectin. **B**: Representative trace (inward currents from oocytes expressing *Ode*-UNC-29:*Ode*-UNC-63:*Ode*-UNC-38) of the 10 seconds application of the control 100 μM acetylcholine, followed by a 10 minutes application of derquantel and abamectin combination and finally, a 10 seconds application of different pyrantel concentrations in the continued presence of the derquantel and abamectin combination. **C**: Concentration-response plots for pyrantel in the absence (n = 6, red) and presence of 0.1 μM derquantel (n = 4, light purple); 0.3 μM abamectin (n = 5, dark green); 0.1 μM derquantel + 0.3 μM abamectin combination (n = 6, olive green). Results were normalized to 100 μM acetylcholine current responses and expressed as mean ± S.E.M. Inhibition with 0.1 μM derquantel + 0.3 μM abamectin combination was greater than that with 0.1 μM derquantel alone and 0.3 μM abamectin alone. The calculated additive effect for the combination of derquantel and abamectin (broken black) was not statistically different (p > 0.05, paired two-tailed student t-test) from the observed additive effect for the combination of derquantel and abamectin (olive green). **D**: Bar chart showing the mean ± S.E.M of the maximum current responses (*R*_*max*_) for 0.1 μM derquantel (n = 4, light purple); 0.3 μM abamectin (n = 5, dark green); 0.1 μM derquantel + 0.3 μM abamectin combination (n = 6, olive green). *R*_*max*_ for the combination of 0.1 μM derquantel + 0.3 μM abamectin was significantly smaller than *R*_*max*_ for 0.1 μM derquantel alone and for 0.3 μM abamectin alone. * p < 0.05 and *** p < 0.001, unpaired two-tailed student t-test.

For this set of experiments, we tested the combination of 0.3 μM abamectin and 0.1 μM derquantel. We selected 0.3 μM abamectin because it is the reported concentration in *H*. *contortus* following the administration of abamectin at its therapeutic dose of 0.2 μM at 12 hours [27]. 0.1 μM derquantel was selected because the reported concentration is 13 μM at 12 hours in the intestine [28] with an uncertain but lower concentration being present within the worm. Fig 5A and 5B show representative traces for *Ode*-UNC-29:*Ode*-UNC-63:*Ode*-UNC-38 responses to pyrantel in the presence of 0.3 μM abamectin alone, and the 0.1 μM derquantel plus 0.3 μM abamectin combination respectively. Concentration-response plots for pyrantel in the absence and presence of 0.1 μM derquantel alone, 0.3 μM abamectin alone, and the 0.1 μM derquantel and 0.3 μM abamectin combination are shown in Fig 5C. The calculated additive effect of the combination of derquantel and abamectin is also shown in Fig 5C. The observed effect was not greater than the calculated additive effect (p > 0.05) so this combination does not show synergism. S3 Table shows the *EC*_*50*_ and *R*_*max*_ values for these experiments. There was no significant difference (p > 0.05) between *EC*_*50*_ values for pyrantel in the absence and presence of derquantel alone, abamectin alone or the derquantel and abamectin combination. However, as before the *R*_*max*_ values for pyrantel in the presence of 0.1 μM derquantel alone, 0.3 μM abamectin alone, and 0.1 μM derquantel and 0.3 μM abamectin combination were significantly smaller (p < 0.001) than the *R*_*max*_ for pyrantel in the absence of derquantel and/or abamectin. Thus both derquantel and abamectin show non-competitive antagonism on the *Ode*-UNC-29:*Ode*-UNC-63:*Ode*-UNC-38 receptor. Fig 5D also shows that *R*_*max*_ values for pyrantel in the presence of 0.1 μM derquantel alone and 0.3 μM abamectin alone were significantly greater (p < 0.05 and p < 0.001) than *R*_*max*_ in the presence of 0.1 μM derquantel and 0.3 μM abamectin combination. Thus the combination of abamectin and derquantel together increases the antagonism but their combination was not significantly greater than the calculated additive effect.

We used pyrantel as the preferred agonist to study the effects of abamectin and derquantel on the *Ode*-UNC-29:*Ode*-UNC-63:*Ode*-UNC-38 receptor because it is more selective than acetylcholine for this receptor [5]. However, we also tested the effects of abamectin and derquantel with acetylcholine as the agonist instead of pyrantel. We found, as with pyrantel, that a lower concentration of 0.1 μM abamectin was a more potent non-competitive acetylcholine antagonist than 0.3 μM or 1 μM abamectin, S1 Fig. We also found that 0.3 μM derquantel acts as a non-competitive antagonist of acetylcholine and that the combination of derquantel and abamectin shows additive inhibitory effects without demonstrating synergism, S2 Fig.

## Discussion

### Abamectin as a non-competitive antagonist of *Ode*-UNC-29:*Ode*-UNC-63:*Ode*-UNC-38

Abamectin, a mixture of avermectins B1a and B1b (Fig 1), belongs to the macrocyclic lactone class of anthelmintics which have been shown to act on GluCls [11]. In addition to the effect of abamectin on GluCls, Puttachary et al [21] showed, using muscle contraction and current-clamp electrophysiological techniques, that abamectin acts as a non-competitive antagonist of *Ascaris suum* (Clade III nematode) somatic muscle nAChRs. In this paper we were interested to see if abamectin had similar effects on the expressed *O*. *dentatum* (Clade V nematode) receptor, *Ode*-UNC-29:*Ode*-UNC-63:*Ode*-UNC-38. Interestingly, we found that abamectin was also a potent non-competitive antagonist of this receptor (Fig 3B).

### Bi-phasic effects of abamectin on *Ode*-UNC-29:*Ode*-UNC-63:*Ode*-UNC-38

The inverse dose-dependent bi-phasic effects of abamectin on *Ode*-UNC-29:*Ode*-UNC-63:*Ode*-UNC-38 suggest that abamectin has two allosteric sites of action on this receptor: one, a higher affinity negative allosteric modulator (NAM) site causing antagonism; and the other, with lower affinity, a positive allosteric modulator (PAM) site, causing a reduction in the antagonism (Fig 4D). The NAM and PAM sites of action of abamectin on the receptors may be in the outer lipid phase of the membrane [29] between the TM1 transmembrane domain of one receptor subunit and the TM3 domain of the adjacent receptor subunit [30]. Since the receptor channel is composed of heterogeneous subunits, binding between one pair of subunits may stabilize closing (negative allosteric modulator) while binding between another pair of subunits may stabilize opening (positive allosteric modulator).

### Derquantel and abamectin combination produces greater effects than either derquantel or abamectin used alone

The effects of a combination of derquantel and abamectin on a mixture of *in situ A*. *suum* somatic nAChR subtypes have been described by Puttachary at al [21]: when derquantel was used alone, *A*. *suum* somatic nAChRs were antagonized in a competitive manner (right-shift in *EC*_*50*_, but no change in *Rmax*); when abamectin was used alone, *A*. *suum* somatic nAChRs were antagonized in a non-competitive manner (reduction in *Rmax*, but no change in *EC*_*50*_); when derquantel and abamectin were used in combination, abamectin potentiated the antagonism of derquantel. In our experiments with *Ode*-UNC-29:*Ode*-UNC-63:*Ode*-UNC-38, derquantel showed non-competitive (Fig 5C) rather than competitive antagonism. With the combination of derquantel and abamectin together, the *R*_*max*_ was significantly smaller than for derquantel alone and abamectin alone (Fig 5D). Additionally, the effects of the combination were greater than those of 0.1 μM derquantel at pyrantel concentrations ≥ 0.3 μM suggesting that the use of abamectin and derquantel in combination is likely to be more effective than either one of the two drugs administered alone.

## Conclusion

Our observations reveal that abamectin, in addition to its recognized effects on GluCl channels also has effects on an UNC-29: UNC-63: UNC-38 subtype of nematode nAChR and that the antagonism is non-competitive with a bi-phasic inverse dose-dependent effect characteristic of two sites of action. The higher affinity site, a negative allosteric site, produces more closing of the nAChR in contrast to the other lower affinity site, a positive allosteric site that produces more opening. The significant observation is that lower concentrations of abamectin produced greater inhibition of the nAChR than higher concentrations. The combination of derquantel and abamectin as Startect^R^ can produce inhibition of nematode worm movement and feeding. Derquantel and abamectin inhibits the nAChRs; and in addition, abamectin inhibits movement and feeding by activating GluCls. The double action of the combination of derquantel and abamectin has the potential to slow resistance development in nematode parasites by acting at a number of different sites of action. The emergence of resistance in numerous nematode parasite species makes the control of helminth infections with the currently available anthelmintics more difficult but the recent introduction and careful use of appropriate anthelmintic combinations may be used to reduce the rate of development of anthelmintic resistance. It should also be noted that effects of abamectin does not involve a simple mode of action and that there are effects on more than one LGIC in addition to the GluCls.

## Supporting Information

S1 Fig Concentration-response plots for acetylcholine in the absence (n = 4, black) and presence of 0.1 μM abamectin (n = 4, dark blue), 0.3 μM abamectin (n = 4, dark green) and 1 μM abamectin (n = 3, pink). Results were normalized to 100 μM acetylcholine current responses and expressed as mean ± S.E.M. The standard errors are smaller than the symbols and some are not visible. Notice 0.1 μM abamectin caused an inhibition of current responses to acetylcholine, and this inhibition was reduced with 0.3 and 1 μM abamectin.
(TIFF)

S2 Fig Concentration-response plots for acetylcholine in the absence (n = 4, black) and presence of 0.3 μM derquantel (n = 4, light purple); 0.1 μM abamectin (n = 4, dark blue); 0.3 μM derquantel + 0.1 μM abamectin combination (n = 4, olive green). Results were normalized to 100 μM acetylcholine current responses and were expressed as mean ± S.E.M. The standard errors are smaller than the symbols and some are not visible. Inhibition with 0.3 μM derquantel + 0.1 μM abamectin combination was greater than that with 0.3 μM derquantel alone and 0.1 μM abamectin alone. The calculated additive effect (_*_) is also plotted.
(TIFF)

S1 Table *EC*_*50*_, *R*_max_, *n*_*H*_ and n numbers for acetylcholine and pyrantel on *Ode*-UNC-29:*Ode*-UNC-63:*Ode*-UNC-38. (DOC)

S2 Table *EC*_*50*_, *R*_max_, *n*_*H*_ and n numbers for pyrantel in the absence and presence of abamectin on *Ode*-UNC-29:*Ode*-UNC-63:*Ode*-UNC-38. (DOC)

S3 Table *EC*_*50*_, *R*_max_, *n*_*H*_ and n numbers for pyrantel in the absence and presence of derquantel, abamectin, and a combination of derquantel and abamectin on *Ode*-UNC-29:*Ode*-UNC-63:*Ode*-UNC-38. (DOC)

S4 Table *EC*_*50*_, *R*_max_, *n*_*H*_ and n numbers for acetylcholine in the absence and presence of 0.1 μM, 0.3 μM and 10 μM abamectin on *Ode*-UNC-29:*Ode*-UNC-63:*Ode*-UNC-38. (DOC)

S5 Table *EC*_*50*_, *R*_max_, *n*_*H*_ and n numbers for acetylcholine in the absence and presence of derquantel, abamectin, and a combination of derquantel and abamectin on *Ode*-UNC-29:*Ode*-UNC-63:*Ode*-UNC-38. (DOC)
