# Supplementary figures and images for: Curiouser and Curiouser: The Macrocyclic Lactone, Abamectin, Is also a Potent Inhibitor of Pyrantel/Tribendimidine Nicotinic Acetylcholine Receptors of Gastro-Intestinal Worms
Source: PLoS One. 2016 Jan 11;11(1):e0146854. doi: 10.1371/journal.pone.0146854 (PMC4709073; doi:10.1371/journal.pone.0146854)

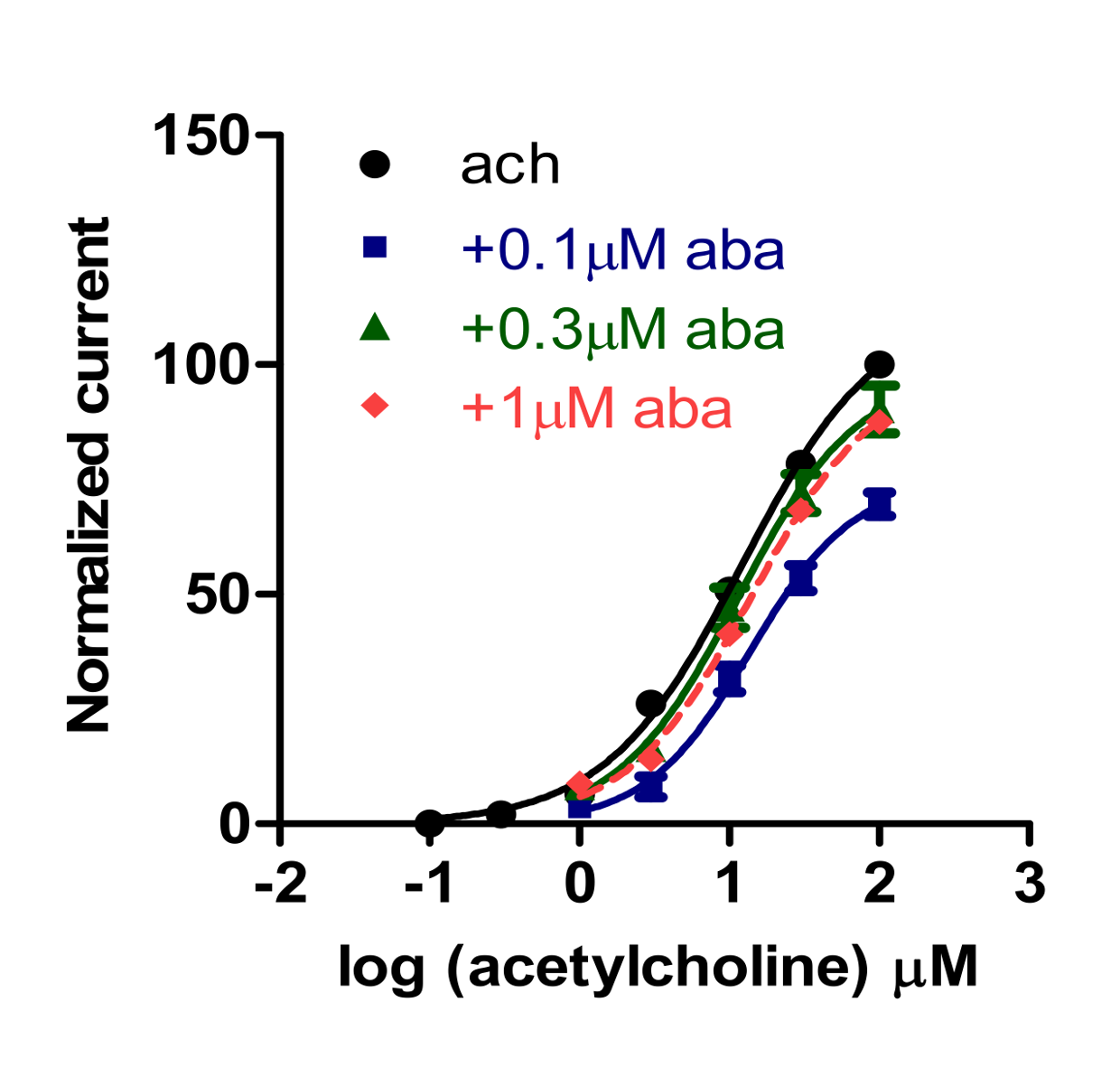

Supplement: S1 Fig — Results were normalized to 100 μM acetylcholine current responses and expressed as mean ± S.E.M. The standard errors are smaller than the symbols and some are not visible. Notice 0.1 μM abamectin caused an inhibition of current responses to acetylcholine, and this inhibition was reduced with 0.3 and 1 μM abamectin. (TIFF) [file pone.0146854.s001.TIFF]

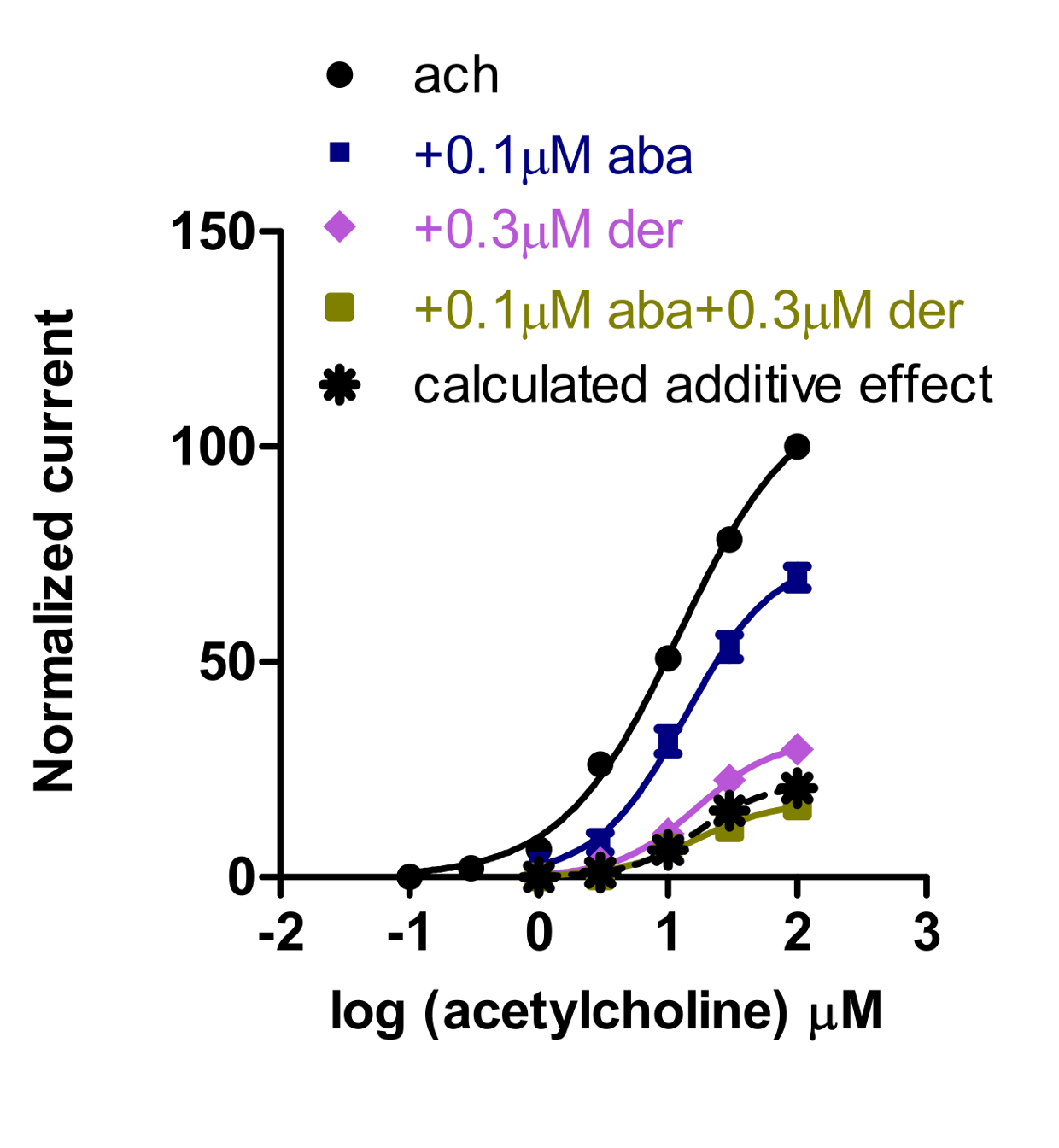

Supplement: S2 Fig — Results were normalized to 100 μM acetylcholine current responses and were expressed as mean ± S.E.M. The standard errors are smaller than the symbols and some are not visible. Inhibition with 0.3 μM derquantel + 0.1 μM abamectin combination was greater than that with 0.3 μM derquantel alone and 0.1 μM abamectin alone. The calculated additive effect (*) is also plotted. (TIFF) [file pone.0146854.s002.TIFF]
