# Supplementary material for: Curiouser and Curiouser: The Macrocyclic Lactone, Abamectin, Is also a Potent Inhibitor of Pyrantel/Tribendimidine Nicotinic Acetylcholine Receptors of Gastro-Intestinal Worms
Source: PLoS One. 2016 Jan 11;11(1):e0146854. doi: 10.1371/journal.pone.0146854 (PMC4709073; doi:10.1371/journal.pone.0146854)
Supplement: S2 Table — (DOC) [file pone.0146854.s004.doc]

| **Drug** | ***EC_50_***  **(mean ± S.E.M, μM)** | ***R_max_***  **(mean ± S.E.M, % 100 μM acetylcholine response)** | ***n_H_*** | **n** |
| --- | --- | --- | --- | --- |
| **Pyrantel** | 0.4 ± 0.0 | 135.5 ± 7.9 | 1.2 ± 0.1 | 6 |
| **Pyrantel + 0.03 μM abamectin** | 0.4 ± 0.0 | 107.3 ± 4.7 | 0.9 ± 0.1 | 5 |
| **Pyrantel + 0.1 μM abamectin** | 0.3 ± 0.0 | 80.6 ± 8.4 | 1.2 ± 0.2 | 6 |
| **Pyrantel + 0.3 μM abamectin** | 0.3 ± 0.1 | 98.4 ± 6.5 | 1.0 ± 0.1 | 5 |
| **Pyrantel + 1 μM abamectin** | 0.3 ± 0.1 | 108.0 ± 3.5 | 1.2 ± 0.1 | 6 |
| **Pyrantel + 10 μM abamectin** | 0.4 ± 0.1 | 106.0 ± 9.4 | 1.5 ± 0.4 | 5 |
