# Supplementary material for: Curiouser and Curiouser: The Macrocyclic Lactone, Abamectin, Is also a Potent Inhibitor of Pyrantel/Tribendimidine Nicotinic Acetylcholine Receptors of Gastro-Intestinal Worms
Source: PLoS One. 2016 Jan 11;11(1):e0146854. doi: 10.1371/journal.pone.0146854 (PMC4709073; doi:10.1371/journal.pone.0146854)
Supplement: S3 Table — (DOC) [file pone.0146854.s005.doc]

| **Drug** | ***EC_50_***  **(mean ± S.E.M, μM)** | ***R_max_***  **(mean ± S.E.M, % 100 μM acetylcholine response)** | ***n_H_*** | **n** |
| --- | --- | --- | --- | --- |
| **Pyrantel** | 0.4 ± 0.0 | 135.5 ± 7.9 | 1.2 ± 0.1 | 6 |
| **Pyrantel + 0.1 μM derquantel** | 0.4 ± 0.0 | 69.4 ± 1.1 | 1.1 ± 0.1 | 4 |
| **Pyrantel + 0.3 μM abamectin** | 0.3 ± 0.1 | 98.4 ± 6.5 | 1.0 ± 0.1 | 5 |
| **Pyrantel + 0.1 μM derquantel + 0.3 μM abamectin** | 0.3 ± 0.1 | 60.9 ± 2.3 | 0.9 ± 01 | 6 |
